# Implementing community-based Dried Blood Spot (DBS) testing for HIV and hepatitis C: a qualitative analysis of key facilitators and ongoing challenges

**DOI:** 10.1186/s12889-022-13525-x

**Published:** 2022-05-31

**Authors:** James Young, Aidan Ablona, Benjamin J. Klassen, Rob Higgins, John Kim, Stephanie Lavoie, Rod Knight, Nathan J. Lachowsky

**Affiliations:** 1 Community-Based Research Centre, 1007-808 Nelson Street, Vancouver, BC V6Z 2H2 Canada; 2 Faculty of Health Sciences, Simon Fraser University, 8888 University Drive, Burnaby, BC V5A 1S6 Canada; 3 School of Public Health and Social Policy, University of Victoria, 3800 Finnerty Road, Victoria, BC V8P 5C2 Canada; 4 National Microbiology Laboratory, Public Health Agency of Canada, 1015 Arlington Street, Winnipeg, MB R3E 3R2 Canada; 5 British Columbia Centre On Substance Use, Providence Health Care, 400-1045 Howe Street, Vancouver, BC V6Z 2A9 Canada; 6 Department of Medicine, Faculty of Medicine, University of British Columbia, 317-2194 Health Sciences Mall, Vancouver, BC V6T 1Z3 Canada

**Keywords:** HIV Testing, HCV Testing, Dried blood spot, Community setting, Implementation Research

## Abstract

**Background:**

In 2018, the Community-Based Research Centre (CBRC) invited gay, bisexual, trans, queer men and Two-Spirit and non-binary people (GBT2Q) at Pride Festivals across Canada to complete in-person *Sex Now* surveys and provide optional dried blood spot (DBS) samples screening for human immunodeficiency virus (HIV) and hepatitis C virus (HCV). As there is a lack of research evaluating the implementation of DBS sampling for GBT2Q in community settings, we aimed to evaluate this intervention, identifying key facilitators and ongoing challenges to implementing community-based DBS screening for HIV/HCV among GBT2Q.

**Methods:**

We conducted sixteen one-on-one interviews with individuals involved with the community-based DBS collection protocol, including research staff, site coordinators, and volunteer DBS collectors. Most individuals involved with DBS collection were “peers” (GBT2Q-identified). The Consolidated Framework for Implementation Research (CFIR) guided our data collection and analysis.

**Results:**

Interviewees felt that DBS collection was a low-barrier, cost-effective, and simple way for peers to quickly screen a large number of *Sex Now* respondents. Interviewees also noted that the community and peer-based aspects of the research helped drive recruitment of *Sex Now* respondents. Most interviewees felt that the provision of results took too long, and that some *Sex Now* respondents would have preferred to receive their test results immediately (e.g., rapid or point-of-care testing).

**Conclusion:**

Peer-based DBS sampling can be an effective and relatively simple way to screen GBT2Q at Pride Festivals for more than one sexually transmitted and blood borne infection.

**Supplementary Information:**

The online version contains supplementary material available at 10.1186/s12889-022-13525-x.

## Background

Canada has joined global efforts to eliminate human immunodeficiency virus (HIV) and hepatitis C virus (HCV) as public health threats by 2030 [1, 2]. However, an estimated 13% of Canadians living with HIV and approximately 44% of Canadians living with chronic HCV are unaware of their infection [3, 4]. While there has been a general decrease in HIV incidence in Canada among gay, bisexual, and other men who have sex with men (gbMSM), particularly since Health Canada’s approval of pre-exposure prophylaxis (PrEP), gbMSM continue to represent the largest proportion of all new adult HIV cases (39.7%) [5]. With respect to HCV, HIV-negative gbMSM have a slightly higher prevalence of HCV and gbMSM living with HIV have a significantly higher prevalence when compared to the Canadian population [6, 7]. Consequently, HIV and HCV epidemics persist in Canada, with both viruses disproportionately affecting gbMSM.

Testing is an essential step in HIV and HCV care, facilitating diagnosis, linkage to treatment, and viral suppression (HIV) or elimination (HCV). In Canada, a variety of testing modalities exist across provincial health jurisdictions. Novel testing modalities can address known barriers to phlebotomy and/or clinic-based testing, such as stigma, discrimination, and inaccessibility (e.g., distance to clinic, wait times) [8]. For example, to reduce the test result wait time required in traditional phlebotomy, in 2005 Health Canada approved an HIV point-of-care testing (POCT) device, where results are returned minutes after the test is conducted [9]. Peer-based methods and testing that occurs outside clinical settings have also been explored, such as community-based POC HCV testing for people who inject drugs [10], at-home HIV or HCV self-testing kits [11], and dried blood spot (DBS) sampling, which involves the collection of blood on filter paper via a finger prick, which is then air dried prior to being transported and screened [12]. DBS sampling can be conducted by non-healthcare professionals and in non-clinical settings [13].

While HIV and HCV testing methods have expanded, “second generation” bio-behavioural surveillance methods have also been introduced to understand better the needs of key populations, such as gay, bisexual, trans, queer men and Two-Spirit and non-binary people (GBT2Q). *Sex Now* is a national periodic behavioural surveillance survey with deep community roots conducted by the Community-Based Research Centre (CBRC) to promote the health and wellness of GBT2Q [14, 15].

In 2018, *Sex Now* was conducted by the CBRC at 15 Lesbian, Gay, Bisexual, Trans, Queer and Two-Spirit (LGBTQ2 +) Pride festivals in six provinces across Canada from June to September of 2018. GBT2Q aged 15 years or older and living in Canada were invited to complete anonymous paper-and-pen health surveys in English or French. One of the primary objectives of *Sex Now 2018* was to understand the proportion of the GBT2Q community with undiagnosed HIV/HCV, helping to inform public health policy. Thus, all *Sex Now* respondents (regardless of HIV/HCV status) who were at least 18 years of age could also optionally provide DBS samples for serological HIV and HCV testing. While DBS testing was not confirmatory (e.g., if a DBS sample was reactive, additional testing was required to provide a formal diagnosis), it was the preferred testing method for *Sex Now 2018* because it could be conducted by non-healthcare professionals and required less equipment and space to collect and store specimens than phlebotomy. Furthermore, DBS collection was less resource- and time-intensive than POCT as it did not require the provision of on-the-spot results or counselling in the event of a reactive test.

For each Pride festival location, the CBRC worked with and provided funding to local community-based partner organizations who identified a primary site coordinator responsible for managing *Sex Now 2018* data collection at their community events. Each site was provided target recruitment numbers for DBS sampling, and each site coordinator was responsible for recruiting and training volunteers. On the day(s) of each Pride event, DBS collectors, which included available CBRC research staff, site coordinators, and volunteers, assisted with the collection of *Sex Now 2018* surveys and DBS samples. Notably, the majority of DBS collectors were “peers” (GBT2Q-identified) and were not healthcare professionals, which aligns with calls for peer-based work and responses.

At each event, after completing the *Sex Now* survey, respondents optionally lined up to provide DBS samples. *Sex Now* respondents were either paired one-on-one with DBS collectors or they could also choose to self-collect their sample. *Sex Now* respondents who wished to receive the results of their HIV/HCV screening tests were asked to provide contact information (phone number or email) for CBRC staff to follow-up via e-mail, telephone, or text message. *Sex Now* respondents were informed that results were not diagnostic, that confirmatory testing should be sought through local public health mechanisms, and that receipt of results could take at least two months from the time of collection. Referrals to local sexually transmitted and blood-borne infection (STBBI) testing options were provided to all interested *Sex Now* respondents. DBS collection cards were batch-shipped at the end of each Pride festival to the Public Health Agency of Canada National Laboratory for HIV Reference Services for screening. A CBRC staff member returned all results concordant with *Sex Now* respondents’ self-reported HIV and/or HCV status through text message or e-mail. All discordant results were returned via telephone, and locally relevant information on appropriate confirmatory testing services were provided to support linkage to diagnosis and care.

Despite second generation surveillance of GBT2Q in Canada historically using DBS to screen for HIV, HCV, and syphilis, there is no published qualitative research evaluating the implementation of DBS sampling for GBT2Q in North American community settings. [16, 17]. Previous research such as the study by Prinsenberg et al., however, have found that at-home DBS self-collection for HCV detection was feasible for gbMSM [18]. There is also minimal data evaluating the collection of DBS samples by non-healthcare professionals. Outside of Canada, Belza et al. and Sikombe et al. found that non-healthcare professionals could be effectively trained to conduct DBS testing, but in one study DBS samples were collected under direct supervision, and both studies provided little qualitative data related to the sample collection process [19, 20]. Finally, the implementation of DBS sampling in community settings, particularly those with a large GBT2Q attendance, has not been evaluated. In a brief letter, Flavell et al. described piloting DBS testing at Birmingham Pride in 2013 and reported that DBS sampling was feasible and acceptable in this community setting but provided few details about the implementation [21]. As GBT2Q continue to represent the key population with the most new HIV cases in Canada, and that many people in Canada who are living with HIV and HCV are unaware of their respective infection(s), the implementation of testing methods in settings that may increase accessibility should be evaluated [5]. Furthermore, given that DBS testing only requires a small blood sample and simple storage and shipping requirements, the effectiveness of DBS testing in community settings where space and/or specialized equipment may be limited should be explored [22]. Thus, this study aimed to evaluate the collection of DBS samples among those carrying out DBS screening and other protocol activities at 2018 Pride Festivals across Canada, identifying key facilitators and ongoing challenges to implementing community-based DBS sampling for HIV/HCV testing and returning of results among GBT2Q.

## Methods

We draw on in-depth, semi-structured interviews with individuals involved with DBS collection and other protocol activities for *Sex Now 2018.* We used stratified, purposive sampling to recruit potential participants from various implementation sites and roles, including research staff, site coordinators, and volunteer DBS collectors. Eligible individuals had participated in developing research protocols, collecting DBS samples, or returning results as part of *Sex Now 2018*, and were able to complete an interview in English. A research staff member not connected to *Sex Now 2018* sent a recruitment e-mail to all local site coordinators who were invited to participate and encouraged to forward the e-mail to any eligible participants. Interviewees were offered a $25 CAD honorarium. Ethics approval was provided by the University of Victoria and the University of British Columbia research ethics boards (BC17-487).

Participants provided verbal informed consent prior to data collection. Two trained research assistants, including the first author, conducted approximately 1-h, one-on-one interviews via telephone or in-person from August to October 2019. The second, seventh, and eighth authors used the Consolidated Framework for Implementation Research (CFIR) to develop an initial semi-structured interview guide, with feedback from all co-authors; the CFIR was chosen because it provides a consistent and comprehensive framework that could be easily customized for this study [23]. Interviewees were asked about their knowledge and beliefs about DBS screening for HIV/HCV, implementing the DBS protocol, and their reflections on what worked well and lessons learned. Those who identified as research staff or site coordinators were also asked about their role in developing the DBS protocol.

Interviews were completed until no new themes emerged, indicating data saturation had been reached. Interviews were audio-recorded, transcribed verbatim, and anonymized by the first author. We used both inductive and deductive techniques to conduct coding. Transcripts were first open coded by the first author to inductively identify ideas relevant to DBS implementation. The first author then deductively coded the data using the five CFIR domains (e.g., Intervention Characteristics, Outer Setting, Inner Setting, Characteristics of Individuals, and Process) and relevant constructs within each domain (e.g., Relative Advantage, Knowledge and Beliefs about the Intervention) as a coding framework. Reliability was assessed through intercoder agreement with the second and third authors. Data were analyzed using NVivo by the first author. All co-authors provided extensive feedback during the development of the manuscript, and all methods were performed in accordance with relevant guidelines and regulations (Declaration of Helsinki, Canadian Tri-Council Research Ethics guidelines).

## Results

We interviewed sixteen participants, including eight site coordinators, five volunteers, and three CBRC staff. 5/16 interviewees were involved with DBS implementation in Ontario, 3/16 in Alberta, 2/16 in British Columbia, Nova Scotia, and across multiple sites, and 1/16 in Quebec and Manitoba; CBRC staff were typically involved with implementation at multiple sites. Additionally, sites implemented the intervention over varying time periods (e.g., one day, three days). Where relevant, site-specific findings from the interviews are provided. Results are organized by the five CFIR domains, accompanied by supporting interviewee quotations and the interviewee’s role in Table 1.

**Table 1:** CFIR Domains & Constructs with Accompanying and Illustrative Quotes

Domain	Construct	Exemplar Quotes
**1. Intervention Characteristics**	1. Complexity	I think it is a very easy procedure. It could be done by anybody. It could be done by the person themselves. It could be done by a peer. It could be done by a volunteer. So, there is very little barrier on who can do this, and how they can do this and the amount of training that they have to go through. It’s very convenient in that regard. (Site Coordinator)
	2. Adaptability	I think you could have even achieved both things. The researchers fundamentally wanted their dried blood spot, while we have a finger pricked I don’t think it would have been difficult to do a Point-of-Care as well… I think that might have made people a little bit happier on the spot. (Volunteer)
	3. Relative Advantage	A. I think it was for the most part it was pretty low barrier and like not as intimidating. Even people who, just anecdotally would come up and, I was helping with recruitment so just hearing people say ‘I’m kind of afraid of needles but I’m going to do it’. Because we could go through and say, it’s not a full vial of blood from your arm, it’s a little finger prick. (Site Coordinator) B. I think it was honestly very beneficial in having community members volunteer and be trained to be able to do the dried blood spot because then it made their friends come, and friends of their friends and people that we might not have ever seen if we had the health professionals leading and doing that work. I think the fact that it felt more community-led and -owned was also something that encouraged a lot of folks to come and get their dried blood spot taken. (Site Coordinator)
	4. Cost	I thought that it was really good because if you’re going to have to pay healthcare professionals it’s going to cost you a fortune and there goes all your funding out the window, where it’s something that someone like me [a non-healthcare professional] can do so long as I have training. To me it’s a good, cost effective way to get it done. (Volunteer)
**2. Process**	1. Engaging	A. I think that the biggest motivation was the overall goal of the study… to help address blood donation. That was probably the biggest, the absolute biggest impetus for me. (Interviewee 14, Site Coordinator) B. Even before I worked for CBRC, I participated in the online Sex Now survey over the last couple of iterations because as a gay man I know that there’s not enough data about us out there and if we don’t participate, there never will be. (CBRC Staff)
	2. Executing	A. The day of implementation at the Pride Festival was an incredibly long, tiring and stressful day. I think I worked about 14 h straight…. I was pleasantly surprised with how well things went. We had very few issues with the actual collection process and recruitment process. (Site Coordinator) B. [E]very type of test you add, adds another level of complexity but one of the things that made it a little bit more challenging was when DBS results… were indeterminate for whatever reason. Like if not enough blood had been collected, or if there was a technical issue with the dried blood spot card, and sometimes the HIV result was sufficient but the Hep C wasn’t, and sometimes the Hep C was and the HIV wasn’t, sometimes they both weren’t sufficient, so for each little complication it just creates another branch of a complicated tree of how results are given. (CBRC Staff) C. [W]hen I was standing outside the tent and I was speaking to a few people, a few people actually declined to participate because I knew that they were [HIV] positive and I was trying to still encourage them to participate because there’s a whole point was that they were aware of that, but I think there was kind of that concern of, well you don’t want me because I’m going to kind of like taint the sample. (Volunteer)
**3. Characteristics of Individuals**	1. Knowledge and Beliefs about the Intervention	A. Increase in testing opportunities for folks, number one; Low barrier testing, number two; and then like data collection for direct policy change, number three. (Site Coordinator) B. I would be reassuring participants, if you’re testing for your own sake, we’re happy to have you in the survey and we hope you participate but don’t be using this for your testing services. I would tell them to give us their DBS and then go to the [clinic], so you can get your own result right away. I wouldn’t want anyone – especially if they’re concerned or unsure of their status waiting several months for that result. That would certainly not be ideal. (Site Coordinator)
**4. Inner Setting**	1. Available Resources	A. I thought a little bit with training there were some sites that we went out and trained them once and then they went off and did training later… it was kind of like a game of telephone. Some, it was nothing detrimental and nothing catastrophic that we weren’t able to correct as, as we went through additional training or through collection. Umm, but protocol fidelity becomes a challenge if there’s not someone there to monitor it. (CBRC Staff) B. [T]he individual that [partner organizations] would task with Sex Now often expressed feeling overwhelmed, because they had their regular responsibilities on top of the Sex Now responsibilities and then their agency would fund the material costs… in very few cases did the agency actually hire a support for whoever they had tasked with implementing Sex Now within their own agency… ideally, in a perfect world where money’s not an issue, do both, like give the agency money and have a support person attached to the agency or at least the region. (CBRC Staff)
	2. Networks and Communications	It started from going to Manitoba, and after each of these events there was a phone call to make sure that people get to provide that feedback, what worked well for them and what didn’t work well from them. We stayed in the background because we were the last one and we actually learned quite a bit from other sites (Site Coordinator)
**5. Outer Setting**	1. Patients’ Needs and Resources	So also understanding how HIV testing can be quite inaccessible for some people, so I think that was a really great – that we were able to actually offer that option during Pride weekend. And the other thing in terms of the Hepatitis C screening, we also know that access to Queer friendly, gay friendly physicians may not be… they may not be as accessible to certain segments of our community (Site Coordinator)
	2. Other	If people hadn’t been hydrating, it was more challenging to collect a sample […] We had limited shade, we had limited tent space or things like that for participants. (Site Coordinator)

### CFIR construct: intervention characteristics

Interviewees noted several characteristics of the DBS intervention that impacted the implementation process. Regarding the overall complexity of the intervention, most interviewees considered DBS sample collection low barrier (Table 1 – Row 1.1). Despite being relatively straightforward, a few interviewees noted that collecting enough blood to fill all five circles on the specimen collection card was somewhat difficult. While interviewees waited to fill each circle, they typically used the time to connect with *Sex Now* respondents.

Interviewees also reflected on the relative advantage of DBS sampling compared with traditional phlebotomy and point-of-care testing (POCT). One benefit that some interviewees perceived when comparing DBS sampling with POCT was that there is no immediate provision of results. Interviewees felt that providing POCT results, especially reactive ones, would be time-consuming and emotionally laborious in this setting. Furthermore, DBS sampling allowed sites to use volunteer DBS collectors without formal healthcare training; at the time of the study only healthcare professionals were regulated to provide POCT (NB: approvals have since been made to allow non healthcare professionals to conduct HIV POCT and allow HIV POC self-testing).

Conversely, other interviewees noted that some *Sex Now* respondents wanted to receive their test results immediately and would have preferred POCT as an option (Table 1 – Row 1.2). At several sites (e.g., Winnipeg, Manitoba and London, Ontario), POCT for HIV was available from other organizations. If *Sex Now* respondents wanted to receive more immediate test results, they were referred to these organizations, highlighting the adaptability of the intervention to local site needs. When comparing DBS sampling to phlebotomy, interviewees highlighted that DBS took up less space, was simpler to store, and was less intimidating for *Sex Now* respondents (Table 1 – Row 1.3A).

Given that some volunteer DBS collectors were also healthcare professionals, interviewees noted the advantages and disadvantages to using non-healthcare professionals and healthcare professionals in collecting DBS samples, highlighting that both approaches had strengths. Overall, interviewees nearly unanimously celebrated the use of non-healthcare professionals for DBS collection. In addition to the cost-saving benefits (Table 1 – Row 1.4), non-healthcare professionals were typically members of the LGBTQ2 + community (unlike most healthcare professionals), which interviewees highlighted as influencing *Sex Now* respondent’s perceptions about the intervention source as being less clinical and more grassroots, trustworthy, and approachable (Table 1 – Row 1.3B). Interviewees also appreciated that using non-healthcare professionals helped build capacity within LGBTQ2 + communities in informed consent processes, biosafety precautions, and biological specimen collection. As for the benefits of using healthcare professionals, two site coordinators felt they were quicker at DBS sample collection and gravitated to the process more quickly. In addition, multiple volunteers felt that in the rare event that a *Sex Now* respondent felt faint while providing a sample, they preferred having a trained healthcare professional nearby to provide care. Overall, a couple interviewees felt that if non-healthcare professionals were collecting DBS samples, at least one healthcare professional should be present to provide medical assistance and help ensure protocol fidelity.

### CFIR construct: process

Interviewees described how process-related factors impacted intervention implementation. For example, the focus on determining the proportion of undiagnosed HIV/HCV in the GBT2Q community was consistently highlighted as a motivator for *Sex Now* respondents and an engagement strategy for implementation personnel. Many interviewees and *Sex Now* respondents agreed that this information was important for informing public health policy (Table 1 – Row 2.1A). Interviewees were also drawn to the intervention because they viewed it as innovative. A CBRC staff member noted that it was unique that testing results were provided to *Sex Now* respondents as in previous surveillance studies in Canada this had not been the case [16]. Additionally, most interviewees were familiar with and championed the work of the *Sex Now* survey, helping them engage *Sex Now* respondents with the intervention, in part because they recognized the importance of *Sex Now* and data on GBT2Q health (Table 1 – Row 2.1B).

Interviewees also described issues with the planning process, stressing that initial targets for recruitment at each site were too high. With Pride festival events typically taking place over one day, it was stressful for some site coordinators to reach these targets. This was exacerbated by bottlenecks in the DBS collection process, such as not having enough volunteers or physical space to collect samples. The protocol predicted volunteer shortfalls and attempted to reduce its impact on DBS collection by allowing *Sex Now* respondents to collect their own samples after training from a DBS collector, however this option was rarely utilized by *Sex Now* respondents. Those who chose to self-collect samples were often healthcare professionals or had experience collecting finger prick blood samples for other reasons (e.g., regular at-home glucose testing). Limited space was also an issue for self-collection. Although self-collectors were sometimes quicker at completing DBS collection, they still occupied a collection station that biosafety protocols required. Overall, CBRC staff noted that sites that collected DBS samples over multiple days were more successful at hitting target numbers, and avoided collecting samples over one long, stressful day. This was partly dependent on the presence of ancillary Pride events or other events taking place where GBT2Q congregate.

Regarding the execution of the intervention, many interviewees felt that the implementation process was smooth and generally adhered to protocol, but that the collection day was very long (Table 1 – Row 2.2A). Some site coordinators, however, noted minor issues with adhering to protocol or with quality control, such as DBS collectors accidentally touching the sample collection area of a DBS card, again highlighting the importance of a designated role to monitor protocol adherence. For the returning of results, there were various factors that led to delays, including *Sex Now* respondents providing incomplete, illegible, and/or inconsistent contact information, as well as time required by the research team to manually enter and verify paper survey data, and to clean and link datasets. For these issues, interviewees emphasized that *more than one* person at a site should be tasked with monitoring and ensuring protocol fidelity. Additionally, returning results was further hampered by the complexity of communicating multiple screening results (Table 1 – Row 2.2B).

Some interviewees also noted inadequate recruitment of people living with HIV as a challenge. Interviewees perceived that people living with HIV were deterred from participating because they didn’t see a point (e.g., already knew they were living with HIV), they thought they were ineligible, or because of HIV-related stigma (Table 1 – Row 2.2C). Screening for HCV was not enough to overcome these barriers for some people living with HIV.

### CFIR construct: characteristics of individuals

Interviewees also reflected on their knowledge and beliefs about the intervention. Overall, they felt that DBS was an innovative, accessible, convenient, and generally accurate testing method that could shape policy change (Table 1 – Row 3.1A). They also believed that providing DBS collection in a large-scale, public setting helped to normalize and destigmatize testing for STBBIs. This is particularly important given that significant stigma towards HIV/HCV persists, and likely may have prevented some potential *Sex Now* respondents from participating in the research altogether.

While the intervention provided a unique way for many GBT2Q to access information about their HIV and HCV status, CBRC staff noted that the returning of test results was more complicated than anticipated as unforeseen circumstances led to delays in *Sex Now* respondents receiving their results. Furthermore, some interviewees stated that they were anecdotally aware of *Sex Now* respondents that never received their results. This may have been due to illegible written contact information or contact information having changed since the time of the survey.

Given the potential wait time for screening results, many interviewees emphasized the part of the protocol encouraging *Sex Now* respondents to pursue alternative testing options if they wanted their results quickly (Table 1 – Row 3.1B). Interviewees highlighted the importance of timeliness in STBBI testing and stressed the primary goal of the study so that *Sex Now* respondents were not disappointed. Notably, expectations became an issue during the returning of results for some *Sex Now* respondents who were unclear that DBS screening results were *not* confirmatory. According to a few interviewees, when some *Sex Now* respondents received reactive HIV screening results, they may not have understood the possibility of false positives, which further heightened the stress *Sex Now* respondents felt.

### CFIR construct: inner setting

Inner setting factors such as training, biweekly operation meetings, and available resources were highlighted as key factors that affected the implementation of DBS testing. CBRC provided in-person training for site coordinators across the country to carry out DBS testing. Site coordinators generally expressed positive feedback regarding this training, but when it came time for them to train their local DBS volunteer collectors, one CBRC staff noted that incorrect information was sometimes communicated (Table 1 – Row 4.1A). While CBRC staff were present at each collection event to ensure protocol fidelity, it was again noted that having more than one person responsible for this task would be ideal.

To help keep the various implementation sites communicating and aligned, CBRC held biweekly operation meetings leading up to Pride events and throughout the recruitment period. As Pride festivals across Canada are staggered throughout the summer months, this allowed subsequent sites to learn and adapt from previous sites’ successes and challenges (Table 1 – Row 4.2) CBRC also centrally coordinated most of the resources that each community partner organization required to carry out DBS collection, and each community partner organization assigned site coordinators to oversee implementation. As one interviewee described, in practice this sometimes meant an increased workload for existing staff at partner organizations (Table 1 – Row 4.1B). CBRC did not dictate how funds provided to a local site had to be spent.

### CFIR construct: outer setting

Several interviewees noted that a key contributor to the success of DBS testing at Pride was that it addressed an unmet need of many *Sex Now* respondents. Specifically, for *Sex Now* respondents who do not live close to a major urban centre, there can be substantial barriers (e.g., travel costs, time) to getting tested, especially if they would like to attend a clinic specialized in GBT2Q health (Table 1 – Row 5.1). Interviewees noted that peer-based DBS testing provided *Sex Now* respondents a unique opportunity to be tested in a space that felt safer for many than clinics that are less familiar with the GBT2Q population.

Additionally, many interviewees commented on how the weather had significant impacts on the success of DBS testing. While as authors we were challenged when deciding what CFIR domain weather fit into, it was an external factor that led to very different experiences across implementation sites. With the majority of DBS testing taking place in an outdoor setting, cold and/or rainy weather appeared to decrease DBS participation, especially considering that most *Sex Now* respondents had to wait outside with no overhead cover. Furthermore, rain disrupted paper data collection, and cold weather caused *Sex Now* respondents to bleed slower, delaying the collection process. As for warm weather, intense heat from the sun meant that physical space under a tent, which is often a scarce commodity at Pride, was critical for protection from the elements (Table 1 – Row 5.2). Conversely, interviewees that were involved with DBS collection at ancillary Pride events that took place indoors noted the convenience of not having to worry about the weather.

## Discussion

Our study found that DBS screening at Pride for HIV/HCV was a low barrier and relatively straightforward sampling method for DBS collectors with and without formal healthcare training to implement. Furthermore, interviewees felt that DBS sampling was an efficient and cost-effective way to screen large numbers of *Sex Now* respondents quickly, although some *Sex Now* respondents voiced to interviewees that they would have preferred to receive the results of their tests immediately. Our findings also highlighted that community and peer-based research that informs public health policy was a key engagement strategy for many *Sex Now* respondents.

There is limited comparable literature on DBS sampling in community settings. Similar published studies often focus on quantitative results (e.g., assessing HIV or HCV prevalence or incidence), or on qualitative results such as willingness to complete at-home HIV testing using DBS [20, 24–27]. Our findings align well with a study by Saludes et al. that found that DBS testing for HCV was easily implemented in a community setting [27], and with Belza et al. and Sikombe et al., who both reported that non-healthcare professionals could be effectively trained to conduct DBS testing [19, 20]. None of these studies, however, discussed challenges and facilitators to DBS implementation in as rich detail as our current study.

Our findings may help future organizers of community DBS sampling anticipate facilitators and challenges to implementation, especially when recruiting GBT2Q at Pride festivals or similar large events. For example, the staggered nature of Pride weekends across Canada allowed for lessons learned to be applied to subsequent collection events. Implementing DBS collection over multiple days helped alleviate strain on staff and volunteers and also helped to increase recruitment numbers. While self-collection was rarely used, uptake may be improved by providing additional space and designating a DBS trainer to that space to improve accessibility. This could allow the trainer to offer instructions to multiple participants simultaneously thereby increasing the collection rate.

Our findings also highlight the significant impact that weather had on DBS testing; cold and rainy weather hampered recruitment as well as the speed of blood drop formation, highlighting how an indoor setting away from the elements can be a helpful alternative for events that typically take place outside. Additionally, while the study findings support the peer-based use of non-healthcare professionals for DBS collection, interviewees repeatedly highlighted the importance of designating adequate resources to monitoring protocol adherence. DBS collection training could be further reinforced by having an experienced DBS collector review the collection protocol with peers ahead of their shift, helping to prevent deviations from protocol.

With respect to the returning of results, survey quality, manual data entry, and the complexity of returning multiple screening results led to lengthy delays; lab processing issues were not a significant factor. Thus, if screening results will be provided when implementing community DBS sampling in a busy setting such as Pride, our findings highlight the need to establish a comprehensive plan for collecting data and returning results ahead of time; respondent contact information should be collected electronically where possible to avoid issues with legibility and data entry. Furthermore, the plan for returning results should consider respondent’s preferred method of contact (e.g., telephone, text, email), whether each of the test results (e.g., HIV, HCV) are concordant with the respondent’s self-reported status, and what will be communicated if one or multiple test results are indeterminate (e.g., due to technical issues or because not enough blood was collected). Where possible, alternative testing methods such as POCT should be offered so that people who would like to receive their results immediately are able to. This, however, requires trained staff who are able to provide follow-up counselling and linkage to care.

As for DBS collection at Pride events, some interviewees felt that it helped to normalize testing for STBBIs. Given that significant stigma towards HIV/HCV prevents some GBT2Q from getting tested in the first place, STBBI screening at large community events may have the potential to help undo this stigma [28]. The ethical implications of someone being diagnosed via DBS screening at a community event such as Pride, however, are significant. Some individuals who complete HIV/HCV screening at Pride may not be as prepared to receive a reactive screening result. A detailed plan for supporting individuals with reactive results is highly recommended. Our findings also reinforce the importance of being aware of and actively preventing exclusionary practices, such as the inadvertent deterrence of some individuals living with HIV from the *Sex Now 2018* study. They also suggest, however, that many GBT2Q, including those living with HIV, may not be aware of or perceive HCV as a significant risk.

While Pride is a unique and well-situated setting to implement social-justice oriented and community-based interventions, the types of people who *are able to* attend Pride and who *do* attend Pride are not typically representative of the general GBT2Q community (e.g., they are often older, have a higher income, a greater number of sex partners, use more alcohol/substances, and test for HIV more) [29, 30]. Thus, while DBS in this setting can increase access to testing, GBT2Q who are the hardest to reach may still not be reached through DBS sampling at Pride [31]. Finally, our findings may also be applicable for organizers that want to implement community-based DBS testing for other key populations that are affected by HIV and/or HCV. These organizations may also consider mobilizing community through policy-oriented research that directly impacts them. By addressing an issue that the community prioritizes, engagement with the intervention may be more likely to be successful.

Our study has many strengths; it is the first evaluation of community-based DBS implementation for GBT2Q in Canada. Our findings are from a national sample of interviewees representing a diversity of implementation roles (e.g., CBRC staff, site coordinators, volunteers). Additionally, the use of CFIR, an established framework for implementation research, provided a common and consistently used set of constructs to evaluate the intervention. With respect to study limitations, the timing of the interviews (over a year after the intervention) may have impacted the amount of detail interviewees could remember. As individual DBS collectors could opt-in to participating in this study, selection bias may be present and those who had a poor experience with implementing DBS with *Sex Now 2018* may have chosen not to participate in the interviews. Of note, Sex Now 2018 recruited survey respondents using language pertaining to contributing evidence towards ending the discriminatory blood deferral policy for men who have sex with men, which may affect this intervention of DBS sampling for HIV and HCV screening in other contexts or populations. Further, we interviewed a relatively small sample size for this qualitative sub-study, and notably, the perspectives of *Sex Now* respondents on DBS collection (including self-collection) at Pride are not present in this analysis as we did not have consent to re-contact *Sex Now* respondents who provided DBS samples for participation in our study. We also did not interview any people at the laboratory where HIV and HCV analysis took place and where sample quality was assessed. These perspectives are important to examine in future research on the implementation of such interventions. Finally, given recent approvals in Canada of non-healthcare professional administered POC rapid HIV testing and rapid HIV self-testing, future research should examine these testing technologies in large community-based venues.

## Conclusions

 This study provides significant insight into key facilitators and ongoing challenges that affect the implementation of community- and peer-based DBS testing and provision of respondent results among GBT2Q. As Canada continues with its goal to eliminate HIV and HCV as public health threats by 2030, innovative screening methods that increase access to testing are needed. DBS sampling is a proven alternative to phlebotomy and POCT, especially in community settings and/or settings where people may not want their test results immediately. Both HIV and HCV continue to disproportionately impact gbMSM living in Canada, highlighting the continued need for GBT2Q-related HIV and HCV research and interventions [5, 6]. Future studies that continue to investigate the implementation of innovative HIV and/or HCV testing methods will allow for better access to testing, increased linkage to care, and ultimately, the elimination of these public health epidemics.

## Supplementary Information

**Additional file 1.** Sex Now 2018 DBS Qualitative Interview Guide.

## Data Availability

The datasets used and/or analysed during the current study are available from the corresponding author on reasonable request.
