# Supplementary material for: Implementing community-based Dried Blood Spot (DBS) testing for HIV and hepatitis C: a qualitative analysis of key facilitators and ongoing challenges
Source: BMC Public Health. 2022 May 31;22:1085. doi: 10.1186/s12889-022-13525-x (PMC9158154; doi:10.1186/s12889-022-13525-x)
Supplement: Supplementary file 1 — Additional file 1. Sex Now 2018 DBS Qualitative Interview Guide. [file 12889_2022_13525_MOESM1_ESM.docx]

**Appendix C: Sex Now 2018 DBS Qualitative Interview Guide**

Research Objective: To identify successes and opportunities for improvement in implementing a protocol for collecting dried-blood spot samples for HIV and Hepatitis C (HepC) in community-based Pride venues and returning test results

The Consolidated Framework for Implementation Research (CFIR; <https://cfirguide.org/constructs/>) is used for the framework of this evaluation.

**CFIR Domain Descriptions**

**Domain: Characteristics of Individuals**

***Construct: Knowledge and Beliefs about the Intervention***

- Individual attitudes toward and value placed on the intervention as well as familiarity with facts, truths, and principles related to the intervention.

**Domain: Process**

***Construct: Engaging***

- Attracting and involving appropriate individuals in the implementation and use of the intervention through a combined strategy of social marketing, education, role modeling, training, and other similar activities.

Sub-construct: ***Formally Appointed Internal Implementation Leaders***

- Individuals from within the organization who have been formally appointed with responsibility for implementing an intervention as coordinator, project manager, team leader, or other similar role.

***Construct: Executing***

- Carrying out or accomplishing the implementation according to plan.

***Construct: Reflecting and Evaluating***

- Quantitative and qualitative feedback about the progress and quality of implementation accompanied with regular personal and team debriefing about progress and experience.

| Topic Area | Questions | Probes |
| --- | --- | --- |
| 1. Introduction | 1. How were you involved with the dried-blood spot sampling (or DBS) with Sex Now 2018? | - Were you a blood collector, a site coordinator, a research staff member, or did you have a different position? |
| 2. Knowledge and Beliefs about the Intervention | 1. Can you tell me about what motivated you to volunteer/work with Sex Now? 2. What did you think of HIV and Hepatitis C (HepC) testing as part of the study? 3. What did you think of collecting biological samples through dried-blood spots? 4. How do you think DBS HIV/HepC testing might be used in non-research settings? 5. Do you have any thoughts about how results from the HIV and Hep C tests were returned to participants? What would you think about using point-of-care HIV/HepC testing, where you get test results immediately, in this context? | - What did you know about the Sex Now survey before? - Was it useful for us to collect samples for HIV/HepC testing? Why? - What do you think about using volunteers to do this, versus healthcare professionals (e.g., nurses, physicians)? - What about self-collection – how might that work or not work? Who do you think it might work well/not work for? Can you tell me why? - Do you think dried-blood spots were the best way to do HIV/HepC testing at Pride venues? Tell me about that. - Do you have any thoughts about returning results from DBS, where results are not available immediately? For some people, immediate results are a benefit, while others might prefer a bit of time. - When you think about DBS, how do you think that the wait time for results compares with other testing experiences? Can you tell me about more about this? |
| 3. Formally Appointed Internal Implementation Leaders  *If participant was a paid staff (e.g., manager, site coordinator) for Sex Now:* | 1. Can you tell me about the role(s) you played in the DBS process of Sex Now? How did you first hear about this work? | - Can you describe your engagement with the conception of the protocol, if any? - Were there opportunities for you to give feedback after the process was set? If so, can you tell me about it? - Can you describer any instances when you raised questions or concerns as you felt necessary? - In your role, were there ways in which you would have liked to have been involved differently? Tell me about that. |
| 4. Executing | 1. How would you describe the overall experience in your role as an ‘implementer’ with Sex Now? | - Were there challenges you faced in making sure the DBS collection was implemented according to the protocol? Tell me about that. - Was collecting participant information for returning results, using the participant information form, done according to protocol? - Was the process of how HIV/HepC test results would be returned clear to you? - How did you gauge participants’ ability to understand the process of getting their test results? - Can you tell me about your interactions with participants and if they understood the consent process? How was this explained, or not? - Was the returning of results done according to protocol? - How were your interactions with other staff or volunteers who were collecting DBS samples? - Can you describe the context of the day when you were involved in data collection (for example, were there important reflections about the weather, the set-up)? |
| 5. Reflecting and Evaluating | 1. Looking back at your experience, what do you think worked well? 2. Looking back at your experience, what are some lessons learned? | - What do you think were the biggest benefits to carrying out HIV/HepC testing using DBS collection at Pride venues and offering for participants to receive their test results? - How did you deal with challenges that arose during the process? - Is there anything you would change if we were going to do HIV/HepC testing at Pride venues in the future? What different community events might also work or work better? |
| Conclusion | 1. Thanks so much for participating in this interview. Do you think that there is anything we missed in terms of your experiences? Would you have any further recommendations to people who might do this work in the future? |  |
